# Are fish oil omega-3 long-chain fatty acids and their derivatives peroxisome proliferator-activated receptor agonists?

**DOI:** 10.1186/1475-2840-7-6

**Published:** 2008-03-20

**Authors:** Osman ABSM Gani

**Affiliations:** 1 Department of Pharmacology, Institute of Medical Biology, Faculty of Medicine, University of Tromsø, 9037 Tromsø, Norway

## Abstract

**Background:**

Peroxisome proliferator-activated receptors (PPARα, PPARγ, and PPARδ) are physiological sensors for glucose and lipid homeostasis. They are also the targets of synthetic drugs; such as fibrates as PPARα agonists which lower lipid level, and glitazones as PPARγ agonists which lower glucose level. As diabetes and metabolic diseases are often associated with high blood glucose and lipid levels, drugs that activate both PPARα/γ would be a logical approach. But synthetically developed PPARα/γ *dual *agonists and glitazones are showing side effects such as weight gain and edema. Therefore, natural compounds and their close derivatives are focused as future drugs against metabolic diseases.

**Presentation of hypothesis:**

Docosahexaenoic acid and eicosapentaenoic acid, which are the fatty acids abundant in fish oil, are traditionally used against metabolic diseases. These fatty acids act as PPAR agonists that transcript the genes involved in glucose and lipid homeostasis. Present hypothesis suggests that the derivatives of these fatty acids are stronger PPAR agonists than the parent compounds. X-ray structures of PPARs indicate that α or β derivatives of fatty acids would fit into PPARα/γ binding cavity. Therefore, the derivatives will exhibit stronger affinities and activities than the parent compounds.

**Testing of the hypothesis:**

Ligand binding assays and gene transactivation assays should be performed to test the hypothesis. Fluorescence-based methods are advantageous in binding assays, because they were found more suitable for fatty acid binding assays. In transactivation assays, care should be taken to remove contaminants from recombinant proteins.

**Implications of the hypothesis:**

Present hypothesis is framed on the basis of molecular structure of natural PPAR agonists. Small structural changes in the molecular structure of fatty acids have a great influence on activating different PPARs. Therefore, this hypothesis bridges the concept of natural PPAR agonists and the use of structural information in designing new drugs against diabetes and metabolic syndrome. The derivatives may also be used as anti-inflammatory and anticancer agents.

## Background

**Figure 1:**
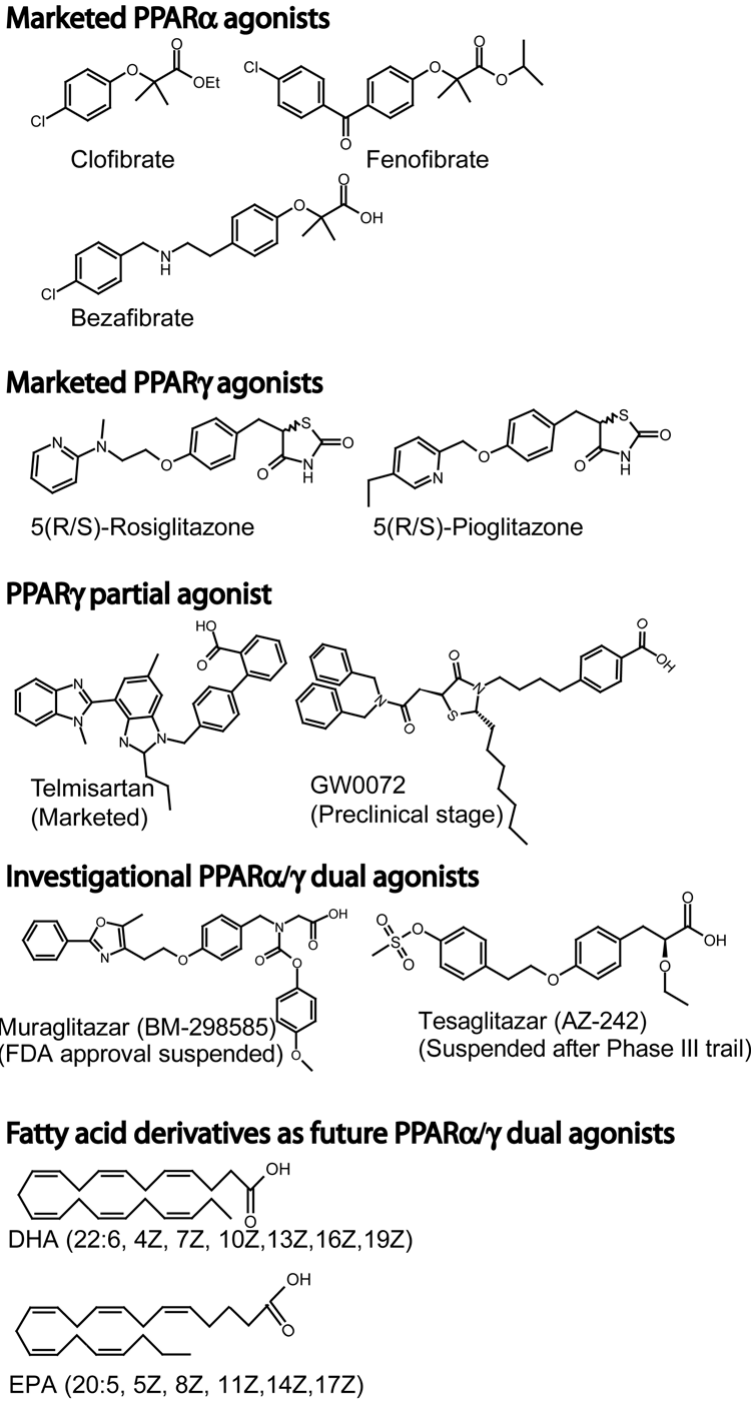
PPAR agonists.

Peroxisome proliferator-activated receptors (PPARs) are members of nuclear receptor superfamily. Three isotypes of PPARs (PPARα, PPARγ, and PPARδ) have distinct tissue distributions, distinct physiological roles, and distinct ligand specificities [1]. Activation of PPARα lowers lipid levels, PPARγ increases insulin sensitivity, and PPARδ regulates cholesterol and glucose levels [1-3]. PPAR ligands comprise natural or synthetic compounds, including fatty acids and eicosanoids [4,5]. As synthetic compounds, fibrates are known as PPARα agonists and glitazones act as PPARγ agonists [2] (Fig. 1). PPARα/γ dual agonists have also been developed, because type 2 diabetes and metabolic syndrome are often associated with high lipid and glucose level in blood.

Antidiabetic glitazones exhibit side effects such as weight gain, edema, and increased risk of myocardial infarctions [6,7], which have limited the use of these drugs in diabetic patients with high lipid levels. The PPARα/γ dual agonists, which reached clinical trials, have been suspended for safety issues [8]. Although much information is not available, these dual agonists are believed to exhibit similar side effects as with glitazones [2,9]. Bezafibrate, a traditional PPARα agonist, has recently been identified as a safe and synthetic pan agonist for all PPAR isotypes [10,11], although with relatively low potency and low affinity (Kd ~5 μM for PPARδ [5]).

Development of drugs from *natural origin *against chronic diseases, such as metabolic syndrome and diabetes, has gain focus recently. Because metabolic system can easily excrete biomolecules and their derivatives, thereby avoid undesirable effects. Fish oil, which contains docosahexaenoic acid (DHA) and eicosapentaenoic acid (EPA), is traditionally used as functional food against metabolic diseases. These beneficial health effects of DHA and EPA are thought to arise from their binding and activating PPARs [12,13]. Phytanic acid, a natural PPAR agonist from human diet, has also been shown to enhance glucose uptake and thereby increases insulin sensitivity, however with less capacity to differentiate adipocytes [14]. A recent study has reported an extract of *Fuligo candida*, Fuligocandin B, to induce 15-deoxy-Δ^12,14 ^prostaglandin J_2 _(15d-PGJ_2_), which is the most potent endogenous PPARγ ligand [15,16].

The hypothesis of this article is drawn on the assumption of PPARα/γ dual agonists from naturally originated molecules, that the derivatives of DHA and EPA would show stronger affinities for PPARs than the parent compounds. Therefore, these compounds may be used as putative drugs against diabetes and metabolic syndrome.

## Presentation of hypothesis

The hypothesis is based on (a) functional roles of PPARs – PPAR agonists reduce blood glucose and lipid levels, and (b) structural details of ligand binding cavity of PPARs, which suggest how a ligand fits into the cavity.

### Functional roles of PPARs

**Figure 2:**
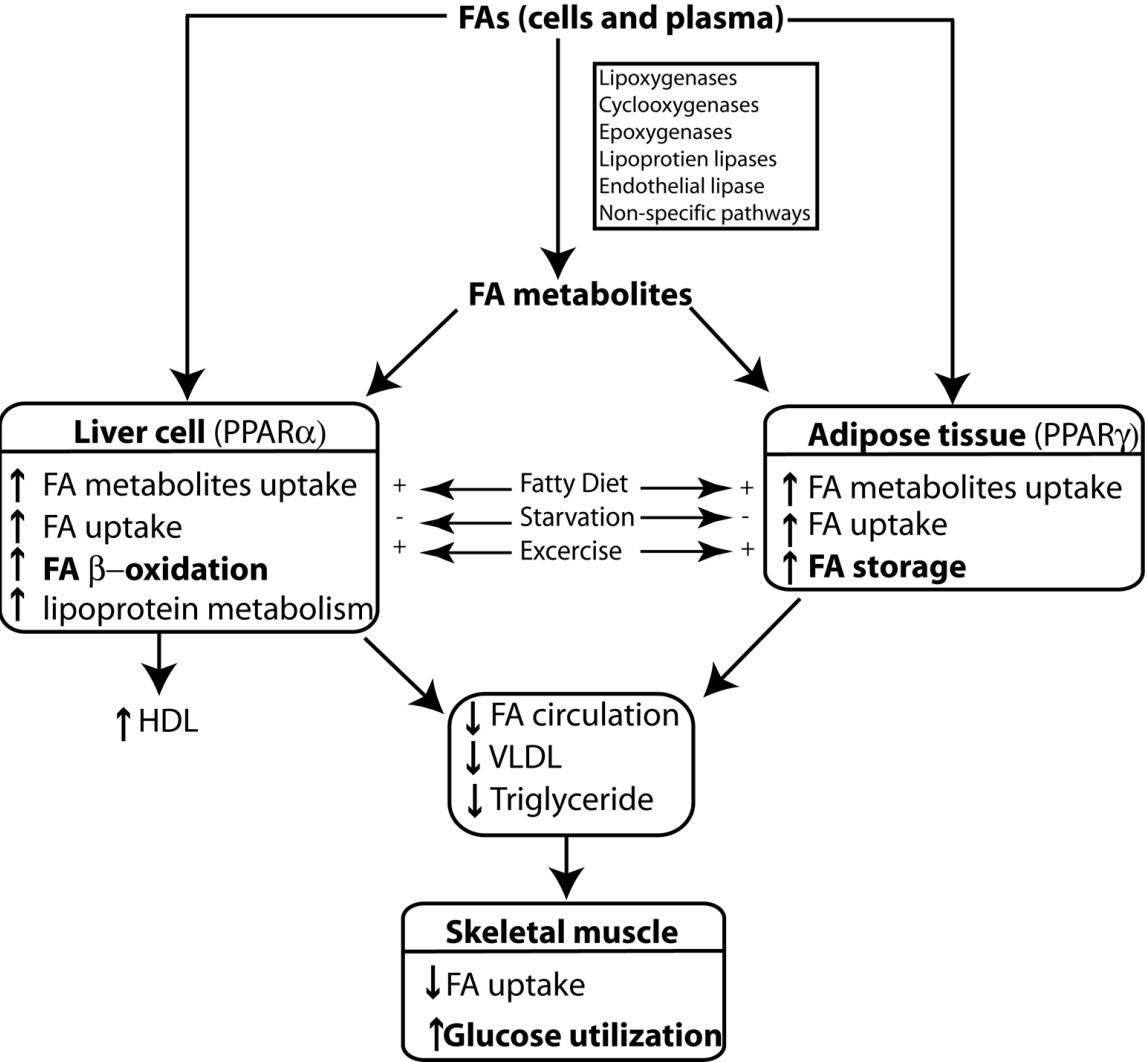
**Complementary actions of PPARα and PPARγ**. PPARα oxidizes fatty acids in the liver cells, and PPARγ stimulates storage of fatty acids in the adipose tissues. Major events are shown in bold fonts. FA = fatty acid; HDL = High-density lipoprotein; VLDL = very low-density lipoprotein.

PPARs perform their activities by endogenous ligands produced by metabolism of fatty acids. Unmetabolized fatty acids can also act as PPAR ligands. Activities of these ligands vary according to their binding specificities for different PPARs, and on distributions of these ligands in different organs [17]. Because of this diversity, not all endogenous PPAR ligands are characterized yet. However, PPAR activities can be summarized as *lipid sensors *by their complementary actions. PPARα is expressed mainly in the tissues with high capacity for fatty acid oxidations, e.g. liver, heart, skeletal muscle etc. On the other hand, PPARγ is expressed predominantly in the adipose tissue but is also expressed in immune and inflammatory cells, mucosa of the colon and placenta. If the fatty acid concentrations are increased, PPARα uptakes and oxidizes fatty acids and their metabolites [18], and PPARγ enhances storage of fatty acids in the adipose tissue [19]. These combined activities of PPARα and PPARγ cause increased utilization of glucose than fatty acids in the skeletal muscles, and cause enhanced insulin sensitivity (Fig. 2). Although PPARδ's role is not well defined, it is also implicated for fatty acid oxidation [20].

### Structure of PPARs

**Figure 3:**
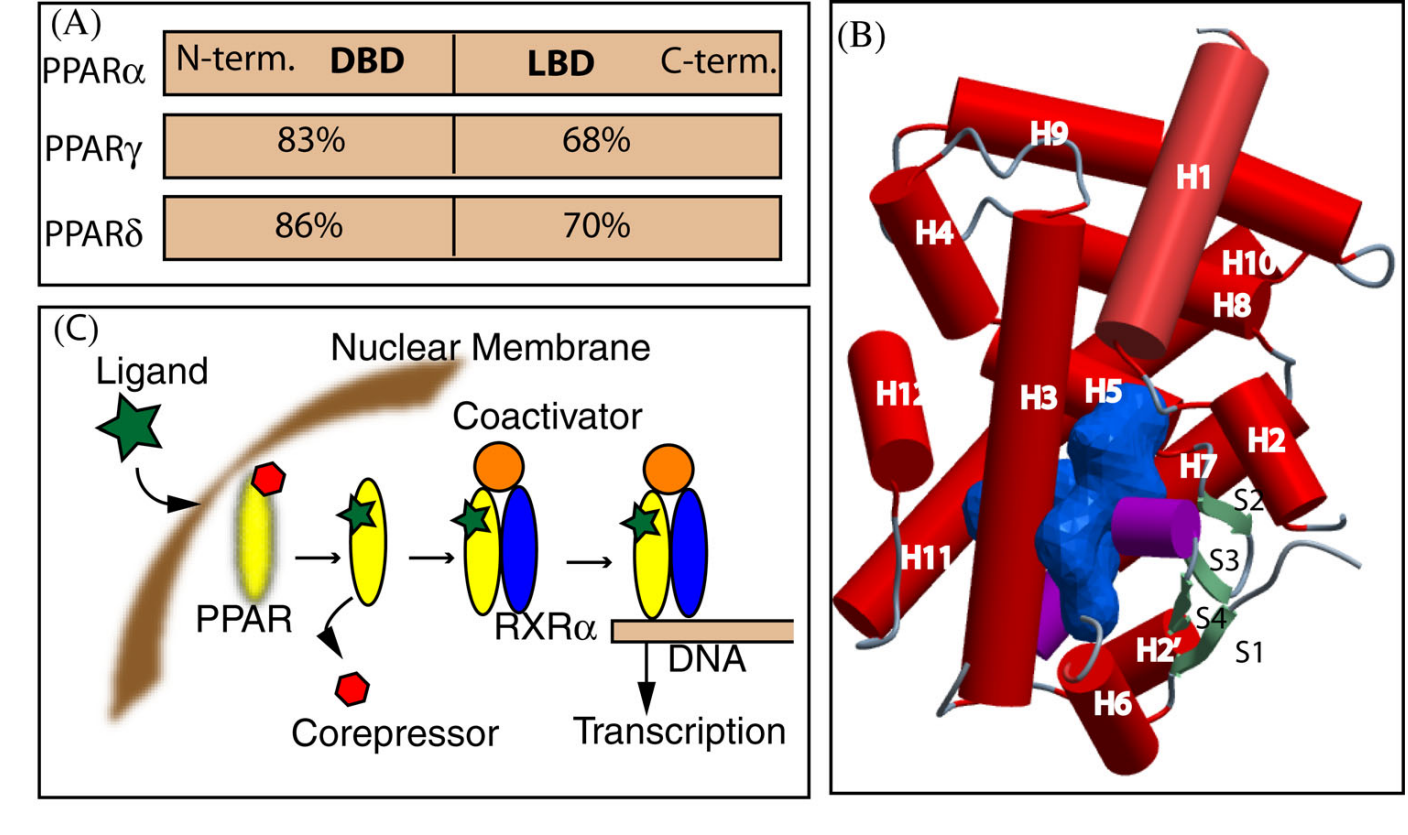
**Structure and functions of PPARs**. (A) Functional domains of PPARs. DBD = DNA binding domain; LBD = ligand binding domain. Numbers denote residue identity in percent compared to PPARα. (B) LBD of PPARs. PPARα-LBD (PDB ID; 1I7G, chain A) is shown with secondary structures; helices in cylindrical form (H: red), β-strands (S: green), 3–10 helix (light blue) and loops (cyan). The blue object shows ligand binding pocket. (C) Ligand-dependent activation of PPARs.

Like other nuclear receptors, 3D structure PPARs consists of a DNA binding domain in the N-terminus and a ligand binding domain (LBD) in the C-terminus [21]. In canonical mechanism, ligand binding to PPARs causes conformational changes in the receptor, which release corepressor and recruit coactivator. Then the receptors form compulsory heterodimers with another nuclear receptor, named retinoid X-receptor (RXR), and the resulting complex finally bind to DNA of target genes [22] (Fig. 3).

To date, 37 X-ray crystallographically determined structures of PPAR-LBDs are deposited in the Protein Data Bank (PDB) in different apo- and holo forms. These structures shows that the LBD is composed of 13 helices and a small four-stranded β-sheet (Fig. 3B). The lower part of LBD contains a Y-shaped cavity with the volume of 1300–1400 Å^3 ^for ligand binding. This cavity includes 34 amino acids; each arm of the cavity is ~12 Å in length. One of the arms is hydrophilic but the other two are hydrophobic. While the amino acids in the polar arm form H-bonds with the ligand polar atoms, those in the hydrophobic arms form non-specific interactions with hydrophobic part of the ligands. Across PPARs, ~80% amino acids in the cavity are conserved, and the overall size of the cavity is also similar [23]. However, the topology is different inside the cavities of different PPARs. These differences influence ligand specificities. Ligand binding stabilizes the LBD in the active conformation; as such coactivator and RXR can bind to the activated LBD [24].

### Fatty acids and their derivatives as PPAR agonists

For centuries, consumption of fatty fish is considered to protect against metabolic diseases. During early 1970s, Danish physicians discovered that Greenland Eskimos consuming fatty fishes exhibited low incidence of heart diseases and arthritis despite high-fat diet. This finding suggested beneficial effects of DHA and EPA. Fish oil supplementation successfully passed clinical trials and is now effectively used for treating metabolic syndrome [25].

Usually, PPAR ligands have three essential parts for optimal binding: (a) polar head group, (b) linker region, and (c) hydrophobic tail. DHA and EPA have a carboxylic group which serves as the polar head group, and their long chains form the required linker and hydrophobic regions. In addition, X-ray structures of PPARs show some free spaces proximal to the polar arm of binding cavity [26], and substitutions at α or β positions on DHA and EPA would fit into these spaces. Specifically, hydrophobic substituents that complement the size of free spaces would be a choice. However, substituents may not fit into PPARδ because of its narrow polar regions [27]. Size and hydrophobic/hydrophilic ratio of substituents may influence the ratio of binding between PPARα and PPARγ [28]. A high PPARα/PPARγ ratio of affinities of putative ligands would be safer dual agonists, because the side effects are thought to arise from high PPARγ affinity [2].

### Limitation of the hypothesis

EPA and DHA have many beneficial health effects which are not typical for PPAR ligands. For example, their capacity to reduce coronary heart disease, blood pressure, primary heart attack and rheumatoid arthritis [29-31] are not observed with typical PPAR ligands. Also, various developmental problems including attention-deficit/hyperactivity disorder have been linked to biological deficiencies in polyunsaturated fatty acids [32]. But these fatty acids do not show many PPAR effects such as reversing insulin resistance [33,34], which may occur due to dissociation between n-3 polyunsaturated fatty acid and lipid metabolism and insulin action in insulin resistant state [35]. Therefore, an interesting observation will be whether substitutions at α or β positions on these fatty acids impart such typical PPAR effects.

## Testing of hypothesis

Ligand binding assays and gene transactivation assays would confirm the hypothesis. Ligand binding assays quantify the binding affinities such as Kd or IC_50 _values. Previously published experiments with fatty acids showed inconsistent binding affinities. Krey et al.[5] reported binding affinities in 5–10 μM range, while Lin et al[36] reported this range of 5–17 nM. The latter range corresponds to the intracellular free fatty acid concentrations which are in the range of 7–50 nM [37]. Until now, no data are available about the affinities of DHA, DHA derivatives, and EPA derivatives. Therefore, fluorescence-based methods as used by Lin et al. may provide correct binding affinities. In transactivation assays of PPARα/γ genes, precautions should be taken to remove unwanted fatty acids in the recombinant proteins [38]. In both ligand binding and transactivation assays, ligands with high ratio of PPARα/γ affinity and activity should be chosen, which mean that compounds showing less PPARγ affinities and activities would be ideal.

## Implication of the hypothesis

The ligand binding cavity of PPARs is 3–4 times larger than the other nuclear receptors, indicating their capability to accommodate and bind variety of natural and synthetic lipophilic acids. Many previous studies have revealed the roles of natural PPAR agonists against specific diseases. For example, natural PPAR agonists such as 15d-PGJ_2 _are emerging as important regulators of immunity and inflammation [39-41]. 15d-PGJ_2 _has also been implicated for antitumor activities [42]. PPARα mediates the anti-inflammatory actions of palmitoylethanolamide, the naturally occurring amide of palmitic acid and ethanolamine [43]. Synthetic PPARγ agonists glitazones have been reported and used in phase I-II human clinical trials as anticancer agents [44].

Present hypothesis is framed on the basis of using natural PPAR agonists that small structural changes in the molecular structure of fatty acids have a great influence on activating different PPARs [45]. Therefore, this hypothesis bridges the concept of natural PPAR agonists and the use of structural information in designing new drugs against diabetes and metabolic syndrome. The derivatives may also be used as anti-inflammatory and anticancer agents.

## Abbreviations

Peroxisome proliferator-activated receptors (PPARs); Docosahexaenoic acid (DHA); Eicosapentaenoic acid (EPA); Ligand binding domain (LBD); Retinoid X-receptor (RXR)

## Competing interests

The author declares no competing interests.
